# Disproportionate Impact of COVID-19 on Breast Cancer Diagnoses in Older Hispanic Women

**DOI:** 10.7759/cureus.89593

**Published:** 2025-08-07

**Authors:** Atharva P Rohatgi, Nathan Tran, Teresa Palkowski, Cheng-I Liao, Kathleen Darcy, Chunqiao Tian, Daniel S Kapp, John K Chan

**Affiliations:** 1 Internal Medicine, Florida Atlantic University Charles E. Schmidt College of Medicine, Boca Raton, USA; 2 Research, Palo Alto Research Center, San Francisco, USA; 3 Obstetrics and Gynecology, University of California, San Francisco (UCSF) Fresno, Fresno, USA; 4 Department of Obstetrics and Gynecology, Kaohsiung Veterans General Hospital, Kaohsiung, TWN; 5 Gynecologic Cancer Center of Excellence Program, Henry M. Jackson Foundation for the Advancement of Military Medicine, Bethesda, USA; 6 Gynecologic Cancer Center of Excellence Program, Henry M Jackson Foundation for the Advancement of Military Medicine, Bethesda, USA; 7 Department of Radiation Oncology, Stanford University School of Medicine, Stanford, USA; 8 Obstetrics and Gynecology, California Pacific Medical Center, San Francisco, USA

**Keywords:** breast cancer, breast cancer screening barriers, cancer epidemiology, covid-19, healthcare disparity

## Abstract

Purpose

This study aimed to evaluate the impact of COVID-19 pandemic on discrepancies between expected and actual breast cancer diagnosis.

Methods

Data on breast cancer incidence were obtained from the United States Cancer Statistics (USCS) program from 2001 to 2020. We compared actual breast cancer incidence rates in the year 2020 to estimated rates based on trends from 2001 to 2019.

Results

From 2001 to 2019, there were 4,005,763 identified cases of breast cancer in the United States. Prior to the pandemic, the incidence rate of breast cancer was 162.87 per 100,000 in females with an annual increase of 1.32% (p < 0.001). A comparison between projected and observed incidences in 2020 revealed a 21,450-case discrepancy, indicating 10.1% fewer diagnosed cases than expected. Patients over 70 years old exhibited the highest discrepancy of more than 13.27% compared to younger cohorts. Hispanic patients saw the largest discrepancy with 13.4% fewer diagnoses compared to only 10.5% in White patients. Intersectional analysis showed that older Hispanic patients aged 65-69 years old residing in the Northeast observed one of the highest discrepancies at nearly 25.9% fewer cases.

Conclusions

The reported breast cancer cases significantly declined during the pandemic. Older age, Hispanic race and ethnicity, and those residing in the Northeast region had the greatest number of missing cases.

## Introduction

The COVID-19 pandemic led to major shifts in healthcare delivery and utilization across the United States. Changes in patient volume and a scarcity of healthcare resources further limited access to care for many individuals [1]. These constraints also disrupted the detection and diagnosis of various diseases, including breast cancer-the most common cancer among women [2,3].

Breast cancer accounts for more than one in 10 cancer diagnoses annually among women in the United States [2]. Previous studies have reported a significant decline in breast cancer diagnoses following the onset of the pandemic, with estimates suggesting reductions in new case identification ranging from 10% to over 20% in 2020 [4-6]. However, while overall declines have been documented, few studies have disaggregated these trends by demographic factors such as age, race/ethnicity, and geographic region. Even prior to the pandemic, disparities in breast cancer incidence, screening access, and outcomes were well-established. For example, non-Hispanic Black women are more likely to be diagnosed at later stages and experience higher mortality rates than their Non-Hispanic White counterparts, despite having similar overall incidence rates [7].

Given these pre-existing inequities, this study aimed to assess the impact of the COVID-19 pandemic on breast cancer diagnoses in the United States by analyzing incidence trends across age groups, racial categories, and geographic regions.

These findings were previously presented as a poster at the 2024 American Society of Clinical Oncology (ASCO) Annual Scientific Meeting on June 2, 2024.

## Materials and methods

Data source

Breast cancer incidence data were obtained from the United States Cancer Statistics (USCS) program for the years 2001 through 2020 [8]. The USCS database integrates data from two major cancer surveillance systems in the U.S.: the Centers for Disease Control and Prevention’s (CDC) National Program of Cancer Registries (NPCR) and the National Cancer Institute’s (NCI) Surveillance, Epidemiology, and End Results (SEER) Program [8]. Together, these programs provide comprehensive, population-based cancer incidence data covering nearly 100% of the U.S. population [8]. Inclusion criteria encompassed all females aged 0 years and older, across all racial and ethnic groups and all U.S. census regions (Northeast, Midwest, South, and West). The final analytic cohort comprised 4,005,763 cases of female breast cancer. Because all data were de-identified and publicly available, the study was considered exempt from institutional review board (IRB) approval.

Study variables

Age at diagnosis was the primary stratification variable and was categorized into five-year intervals for individuals aged 20 to 79 years (e.g., 20-24, 25-29, 30-34, etc.) to facilitate age-specific trend analysis. Individuals aged 0-19 and those aged 80 years and older were categorized separately due to the lower number of cases in these age groups. Based upon the definition set by the National Cancer Institute (NCI), for the purposes of this study and early-onset breast cancer was defined as diagnosis occurring before the age of 50 [9].

Race and ethnicity were categorized into four mutually exclusive groups: non-Hispanic White (NHW), non-Hispanic Black (NHB), Hispanic, and non-Hispanic Asian and Pacific Islander (NHAPI). Geographic location was categorized by U.S. census region: Northeast, Midwest, South, and West. These categorizations allowed for stratified analyses to assess incidence trends across key demographic and geographic subgroups.

Statistical analysis

Statistical analysis was performed using SEER*Stat version 8.4.1.2 and the Joinpoint Regression Program version 5.0.2, developed by the National Cancer Institute (NCI). Breast cancer incidence rates were calculated per 100,000 female population and age-adjusted to the 2000 U.S. standard population to allow for consistent comparisons over time and across groups. Trends in incidence were evaluated using Joinpoint regression analysis, which identifies points where a statistically significant change in trend occurs. For each subgroup, we estimated the average annual percent change (AAPC) in incidence rates over the study period. AAPCs and their 95% confidence intervals were calculated to quantify the direction and magnitude of changes in incidence.

To assess the potential impact of the COVID-19 pandemic on breast cancer detection, we compared observed incidence rates in 2020 to projected rates for that year based on linear trends from 2001 to 2019. Expected 2020 incidence rates were derived from linear trends estimated via Joinpoint regression using data from 2001 to 2019, with 95% confidence intervals used to reflect uncertainty in these projections. Differences between observed and expected incidence rates were examined overall and within each age, race/ethnicity, and regional subgroup. Two-sided p-values < 0.05 were considered statistically significant for all analyses.

## Results

From 2001 to 2019, there were 4,005,763 identified cases of breast cancer in the United States. In 2019, breast cancer incidence was the highest in older aged females at 483.1/100,000 in women aged between 70 and 74, followed by 474.5 and 431.8 in the 75-79 and 65-69 age groups, respectively. Based on race, the incidence was highest in NHW women at 138.4/100,000, followed by NHB women at 131.3. Furthermore, the incidence was the highest in the Northeast at 140.4/100,000, followed by an incidence of 134.3 in the Midwest (Table 1). 

**Table 1:** Breast cancer demographics, incidence, trends, and gap percentage Count (N) (%) refers to the number and percentage of total breast cancer cases recorded in the USCS dataset for each subgroup. Gap % indicates the percentage difference between the observed breast cancer incidence in 2020 and the expected incidence for 2020, which was estimated by projecting the trend from 2001 to 2019. AAPC: average annual percent change; CI: confidence interval; NHW: non-Hispanic White; NHB: non-Hispanic Black; NHAPI: non-Hispanic Asian and Pacific Islander; USCS: United States Cancer Statistics a = average annual percent change (AAPC) from 2001 to 2019, b = observed breast cancer incidence in 2019 (per 100,000 women), c = expected incidence in 2020, based on projection from 2001–2019 trends (per 100,000 women), d = observed incidence in 2020 (per 100,000 women) *p < 0.05, ** p < 0.01, *** p < 0.001

2001-2020	Count (N) (%)	2001-2019 AAPC^a^ (95% CI)	2019 incidence ^b^	2020 expected ^c^	2020 observed ^d^	Gap % (95% CI)
Overall	4,529,724 (100%)	-0.08 (-0.34 to 0.18)	132.00	132.60	119.25	-10.07 (-10.18 to -9.95)
Age Group						
20-24	3,358 (0.1%)	-0.33 (-0.89 to 0.23)	1.79	1.82	1.72	-5.95 (-6.45 to -5.43)
25-29	19,872 (0.4%)	-0.94*** (-1.33 to -0.54)	10.70	10.90	10.42	-4.45 (-4.77 to -4.05)
30-34	59,146 (1.3%)	-4.17*** (-5.64 to -2.67)	30.79	31.27	30.39	-2.8 (-3.4 to -2.22)
35-39	130,185 (2.9%)	2.09*** (1.4 to 2.78)	66.23	66.40	63.54	-4.3 (-4.45 to -4.15)
40-44	264,294 (5.8%)	-4.25*** (-6.16 to -2.31)	133.74	134.49	125.08	-6.99 (-7.12 to -6.87)
45-49	412,948 (9.1%)	0.66** (0.22 to 1.11)	204.72	208.33	190.45	-8.59 (-10.2 to -6.94)
50-54	488,364 (10.8%)	0.15 (-0.85 to 1.15)	244.91	247.43	221.45	-10.5 (-10.97 to -10.03)
55-59	544,083 (12%)	-0.58 (-1.19 to 0.04)	277.74	279.55	253.98	-9.15 (-9.51 to -8.79)
60-64	582,242 (12.9%)	-0.1 (-0.73 to 0.54)	345.23	345.12	308.13	-10.72 (-10.89 to -10.54)
65-69	583,150 (12.9%)	-0.74* (-1.43 to -0.04)	431.79	433.08	382.84	-11.6 (-11.85 to -11.35)
70-74	499,675 (11%)	1.45* (0.16 to 2.75)	483.06	486.95	422.31	-13.27 (-13.69 to -12.85)
75-79	400,362 (8.8%)	-0.74** (-1.18 to -0.3)	474.47	477.69	422.10	-11.64 (-11.76 to -11.51)
80-84	287,769 (6.4%)	0.57 (-0.53 to 1.69)	430.63	444.85	379.25	-14.75 (-18.22 to -11.13)
85+	254,276 (5.6%)	2.51*** (1.6 to 3.44)	326.98	332.66	279.81	-15.89 (-17.99 to -13.73)
Race						
NHW	3,474,109 (76.7%)	-0.08 (-0.33 to 0.17)	138.41	139.12	125.32	-9.92 (-10.03 to -9.8)
NHB	496,054 (11%)	0.63*** (0.38 to 0.88)	131.27	131.47	119.44	-9.15 (-9.53 to -8.76)
Hispanic	336,536 (7.4%)	0.32* (0.05 to 0.6)	101.04	101.92	88.27	-13.39 (-13.71 to -13.07)
NHAPI	164,716 (3.6%)	1.06*** (0.63 to 1.5)	106.84	108.56	94.54	-12.92 (-13.11 to -12.72)
Other or unknown	22,763 (0.5%)	0.79** (0.25 to 1.33)	168.62	173.81	163.16	-6.13 (-7.49 to -4.74)
U.S. Division						
Northeast	907,913 (20%)	0.11 (-0.31 to 0.54)	140.37	141.11	124.76	-11.59 (-11.72 to -11.45)
Midwest	1,003,671 (22.2%)	-0.03 (-0.3 to 0.24)	134.31	135.14	123.12	-8.9 (-9.02 to -8.78)
South	1,645,145 (36.3%)	-0.03 (-0.34 to 0.27)	129.34	129.95	118.03	-9.17 (-9.31 to -9.04)
West	973,420 (21.5%)	-0.39* (-0.73 to -0.04)	127.82	128.13	113.73	-11.24 (-11.38 to -11.09)
Stage						
Local	2,624,136 (67.2%)	0.38* (0.08 to 0.68)	86.11	87.09	76.80	-11.81 (-11.94 to -11.69)
Regional	875,791 (18.9%)	-0.8*** (-0.93 to -0.67)	34.55	34.27	31.68	-7.55 (-7.67 to -7.43)
Distant	158,830 (8.5%)	1.55*** (1.2 to 1.9)	7.46	7.49	7.21	-3.77 (-4.25 to -3.3)

**Figure 1:**
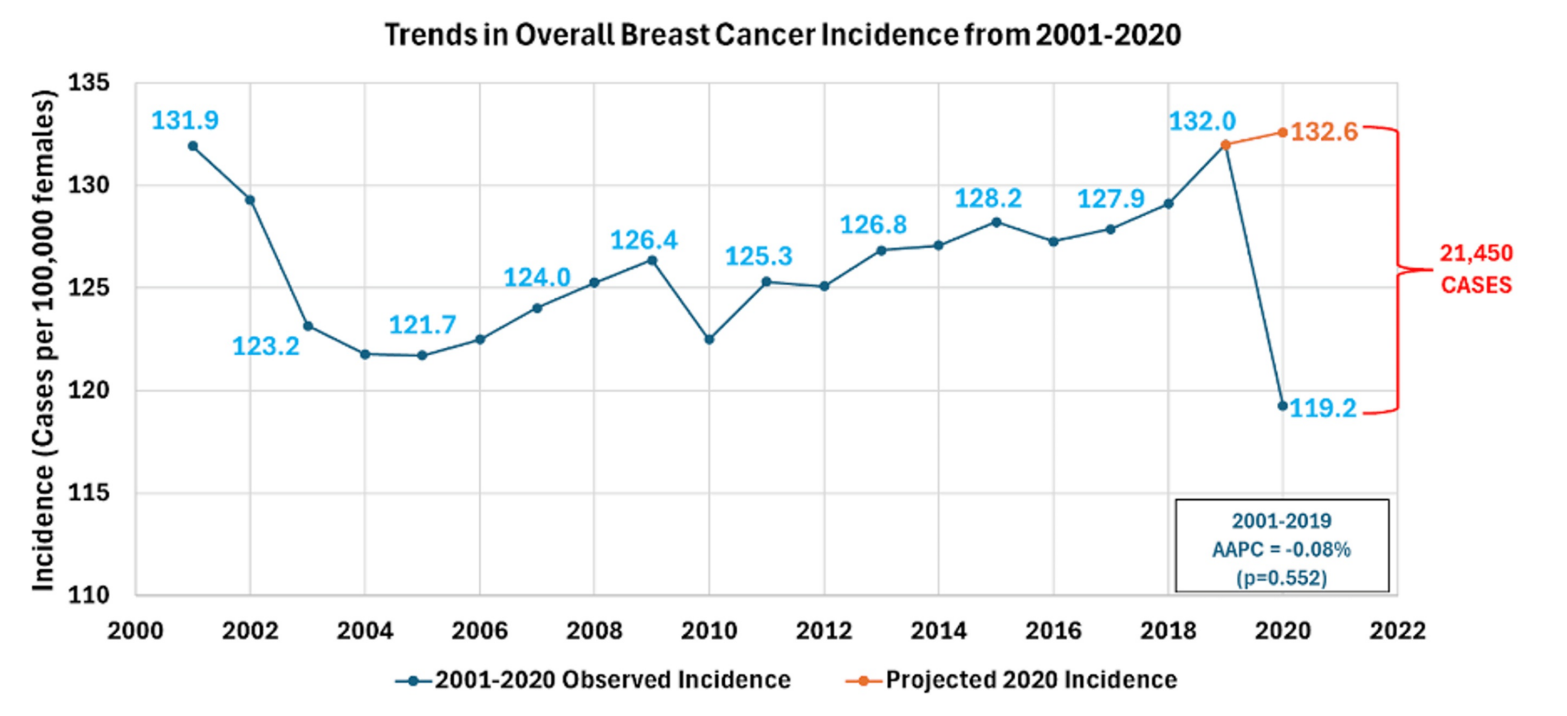
Trends in overall breast cancer incidence from 2001-2020 and 2020 estimated incidence

In 2019, prior to the pandemic, the overall incidence rate of breast cancer was 132.0 per 100,000 in females in the U.S. Using prior trends, it was expected that the incidence of breast cancer to be 132.6 per 100,000 adults (213,092 total cases) in 2020. However, the reported incidence was only 119.3 per 100,000 (191,642). Thus, there were 10.1% (21,450) less breast cancer cases than expected (Figure 1). 

The difference in expected and reported breast cancer diagnoses was most pronounced in older age groups with 15.9%, 14.8%, and 13.3% cases missed for the age groups 85+, 80-84, and 70-74, respectively. Based on racial and ethnic groups, we found that Hispanic females had the greatest number of missed cases at 13.4%, followed by NHAPI females at 12.9%, then 9.9%, and 9.2% of NHAPI, NHW, and NHB females, respectively. The largest discrepancy in missed diagnosis was found in the Northeast at 11.59%, followed by 11.24% in the West. 

Lastly, an exploratory intersectional analysis combining all of these demographic factors revealed that Hispanic women aged 40-44 years old in the Midwest and 65-69 years old in the Northeast were among the highest groups with the largest discrepancy at 27.01% and 25.91%, respectively. By contrast, their White counterparts saw significantly less discrepancies in diagnoses at 3.16% and 9.41%, respectively. 

## Discussion

To our knowledge, this study is one of the first nationwide, population-level analyses to identify specific demographic groups that experienced the greatest declines in breast cancer diagnoses across age, race, and geographic regions during the COVID-19 pandemic. Our findings highlight that the steepest relative gap in cancer incidence occurred among Hispanic and NHAPI women, adults over age 70, and Hispanic populations in the Southern United States. 

Our findings further contribute to previous research that showed overall declines in breast cancer screening and diagnoses during the early months of the pandemic (March-May 2020). For example, studies conducted by Nyante et al. and Sprague et al. both showed significant reduction in the usage of mammography for breast cancer screening during 2020, especially in the initial lockdown period when preventative services were widely paused [4,10-11]. However, these studies were short-term and regional and did not examine national breast cancer incidence trends over a longer time period or by demographic subgroups. Moreover, other studies estimated that 40.9% of adults avoided medical care including preventative due to the pandemic [12]. By contrast, our study utilizes longitudinal, nationwide cancer incidence data from 2001 to 2020 to assess pandemic-related diagnostic gaps across age, race, and region.

Delays in care or decrease in preventative care services caused by the pandemic can have deleterious effects on adults. Care delays have been linked to increased mortality for patients, higher costs, and worse patient outcomes [13-15]. These adverse outcomes associated with care delays are the most damaging for patients who are already in marginalized communities. Minorities have been previously documented to have worse health outcomes and higher mortalities [16-18]. Prior studies have also shown that racial minorities faced greater barriers in accessing healthcare during the pandemic. For example, data from the National Health Interview Survey (NHIS) and other sources indicated that Hispanic and NHAPI women were more likely to report delays in preventative care, loss of employment-linked insurance, and increased food or housing insecurities during the pandemic [12,18-19]. Others might have delays in care due to financial barriers, lack of access to transportation that worsened during the pandemic, and increased need for childcare [20]. Moreover, another study conducted by Fedewa et al. reported that screening mammograms decreased by more than 30% in Asian and Hispanic women in early 2020 compared to a smaller decline in non-Hispanic White women [21]. Our findings are consistent with prior studies; Hispanic and NHAPI women saw greater gaps between expected and actual breast cancer diagnosis compared to non-Hispanic White women. 

Our study also found a more pronounced gap in diagnosis among women aged 70 years and older. This disparity seen in this older age cohort may be due to several factors. For example, older adults had an elevated risk for developing severe illness and mortality from COVID-19 and may have intentionally deferred medical appointments, including cancer screenings due to safety concerns [22]. In addition, the gap in diagnosis rates may reflect the underlying age-specific incidence of breast cancer in the United States; women aged 70-80 years have an incidence rate of approximately 450 per 100,000, nearly double that of women aged 55-59 [23]. Given their higher baseline risk, even modest reductions in screening or healthcare access among older women could lead to disproportionately large declines in diagnosed cases during the pandemic.

Moreover, gaps in diagnosis differed based on region of the U.S. Our analysis based on region showed that Southern states saw some of the greatest relative drops in expected versus actual incidence, especially among Hispanic women. This discrepancy among regions may be possibly attributed to structural and policy-level differences in healthcare systems and public health responses. For example, previous system-level studies have shown that the more poor uninsured adults reside in the South when compared to other regions of the U.S. [24]. Furthermore, this region has higher uninsured rates and a more limited eligibility for Medicaid compared to other regions within the country [25]. In addition, the southern states in the U.S. saw significantly higher cases and deaths associated with COVID-19 when compared to the rest of the country [26,27]. Together, limited insurance availability and higher COVID-19 incidence may have contributed to the larger gaps in breast cancer diagnoses observed during the pandemic in the South. 

Our study has limitations. We were unable to account for individual-level socioeconomic status, comorbidities, insurance coverage, and adherence to screening, all of which are likely confounding variables. We also did not include data on local COVID-19 transmission rates or regional policy measures such as stay-at-home orders, which may have influenced care access and delayed reporting. Furthermore, the dataset only includes reported diagnoses and not underlying screening behavior, so the exact mechanisms for missed diagnoses remain unclear. 

Despite these limitations, our study has several strengths. We utilized a national-level dataset with a large sample size of over four million records representative of the population within the U.S. Unlike many prior analyses, which focused only on mammography volume or short-term trends, we evaluated incidence patterns over two decades and estimated expected diagnoses using Joinpoint regression methods. Our approach is the first to contextualize the observations in 2020 in the setting of the COVID-19 pandemic on the incidence of breast cancer cases and determine the demographic groups most at-risk of missing breast cancer diagnosis. Policymakers and public health agencies should prioritize targeted resource allocation to demographic groups disproportionately affected by potentially underdiagnosed cases of breast cancer during the COVID-19 pandemic. Our findings underscore the need for tailored outreach, enhanced access to screening services, and culturally competent interventions for vulnerable populations. Further studies like ours are warranted to inform health policy and direct resource allocation towards detecting and treating breast cancer cases in vulnerable populations. 

## Conclusions

Our study highlights significant disruptions in breast cancer detection among vulnerable populations during the COVID-19 pandemic. There is a critical imperative to direct targeted resources and implement proactive strategies aimed at populations disproportionately affected by potentially underdiagnosed breast cancer cases during the pandemic. Targeted outreach is essential for the most at-risk individuals, particularly older women, Hispanic and NHAPI populations, and residents of the South. Further research is warranted to understand the different causes of these disparities and evaluate the effectiveness of catch-up screening initiatives and tailored interventions for vulnerable populations.
